# Resolution of Chronic Shoulder Pain after Repair of a Posttraumatic Diaphragmatic Hernia: A 22-Year Delay in Diagnosis and Treatment

**DOI:** 10.1155/2020/7984936

**Published:** 2020-01-06

**Authors:** Brody W. King, John G. Skedros, Robert E. Glasgow, D. Glen Morrell

**Affiliations:** ^1^University of Utah School of Medicine, Salt Lake City, Utah, USA; ^2^Utah Orthopaedic Specialists, Salt Lake City, Utah, USA; ^3^Intermountain Medical Center, Salt Lake City, Utah, USA; ^4^University of Utah Department of Surgery, Salt Lake City, Utah, USA; ^5^Tanner Clinic, Layton, Utah, USA

## Abstract

Diagnosing traumatic diaphragmatic rupture (TDR) due to penetrating rib fractures is challenging because the lesions are often too small to be detected and may present years after injury. Patients with delays in diagnosis of TDR rarely present with orthopaedic-related complaints of pain. We report the case of a 52-year-old female who presented with chronic left shoulder pain following a motor vehicle accident (MVA). In addition to left-side lower rib fractures, she also sustained a left-sided splenic laceration, pneumothorax, and two-part upper humerus fracture. Fracture treatment was percutaneous pinning; the other injuries were treated nonoperatively. Her shoulder pain could not be attributed to shoulder or neck pathology. Twenty years after the MVA, she began experiencing episodes of left-sided abdominal pain and nausea. A CT scan obtained two years later revealed a diaphragm hernia, which was repaired laparoscopically. Unique aspects of this case include (1) presentation to an orthopaedic surgeon with a chief complaint of chronic shoulder pain; (2) at 22 years, this is the fourth longest case of a delay in diagnosis of TDR; and (3) the unique symptom of ipsilateral referred shoulder pain, which immediately improved after hernia repair.

## 1. Introduction

Traumatic diaphragmatic rupture (TDR) is a common, likely underreported, consequence of blunt force trauma or penetrating injuries to the abdomen and chest and is often associated with multiple traumatic injuries [1]. Up to 90% of cases are caused by motor vehicle accidents (MVAs) [2], as was the cause of the case that we report here. Up to 66% of TDRs are not diagnosed during initial workup [3], which is most likely explained by absent, nonspecific, and/or intermittent symptoms that are often delayed in onset [4]. Most TDRs are diagnosed either incidentally or with nonspecific gastrointestinal/pulmonary symptoms. CT imaging can reveal a “collar sign” or a “funky fat sign”, indicating herniation of abdominal viscera or omental fat, respectively [5].

Patients with intermittent, nonspecific symptoms may go undiagnosed for weeks, months, or even many years. Our review of the literature revealed that the majority of delayed diaphragmatic hernia diagnoses occurred within one year of initial injury; however, 17 cases were reported between two and eight years after initial injury [6–14], seven cases between 10 and 20 years [3, 7, 15–19], and three cases over 20 years [20–22]. There have been reports of incidental diagnosis in asymptomatic patients [23]; however, this is uncommon. Most patients present with symptoms of respiratory distress including dyspnea and shortness of breath, abdominal pain, and/or nausea/vomiting [6–22]. We report the fourth longest diagnosis delay at 22 years, with a unique presenting symptom of chronic left-sided shoulder pain, which was why our patient initially sought consultation with an orthopaedic surgeon.

## 2. Case Presentation

The patient is an otherwise healthy 52-year-old female (height 170 cm, weight 66 kg, BMI 22.8) with a long history of left shoulder pain following motor vehicle accident (MVA) 22 years prior. The MVA occurred on June 26, 1996, when she was hit on the driver's side of her car, which she was driving. She sustained multiple left-sided skeletal and visceral injuries including a splenic laceration, pneumothorax, two-part upper humerus fracture, and fractures of the sixth through eleventh ribs. Her initial treatment after the MVA included placement of a chest tube, admission to the intensive care unit for three days, left humerus fracture repair via percutaneous pinning, and nonoperative management of her splenic laceration. The patient was in the hospital for 10 days.

During the next few months, all apparent injuries healed; however, the left shoulder pain persisted and began radiating into the left mandible. The dull pain was never less than a 2/10 and intermittently increased to an 8/10 during cold weather and occasionally after large meals (0 = no pain, 10 = worst pain, determined with the visual analogue scale). Physical therapy and subacromial and glenohumeral corticosteroid injections were given in the first year following injury but were discontinued because there was no improvement in shoulder pain.

Ten years after the MVA, the patient consulted with one of us (orthopaedic surgeon, J.G.S.). CT scans and other radiology images and reports obtained during the initial evaluation following the MVA were unavailable at that time. This consultation was followed by serial orthopaedic physical examinations spanning several months. Possible diagnoses of a rotator cuff tear or glenohumeral injuries (e.g., labrum tear) and other sequelae (e.g., posttraumatic arthritis) were considered as possible causes of shoulder pain; however, the pain could not be recreated with provocative manual tests. Magnetic resonance scanning showed no significant pathology of the neck or shoulder. Cardiac etiologies were also ruled out. Surgery with arthroscopic evaluation was performed and the buried pins were removed from the left upper humerus, but relief of shoulder pain was not achieved.

**Figure 1:**
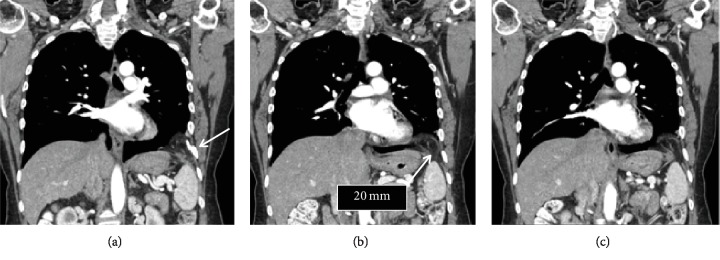
Coronal CT scan images showing diaphragmatic omental herniation (right side of each image). The arrow in (a) shows a healed rib fracture. The arrow in (b) indicates the hernia, which can be seen in each image.

**Figure 2:**
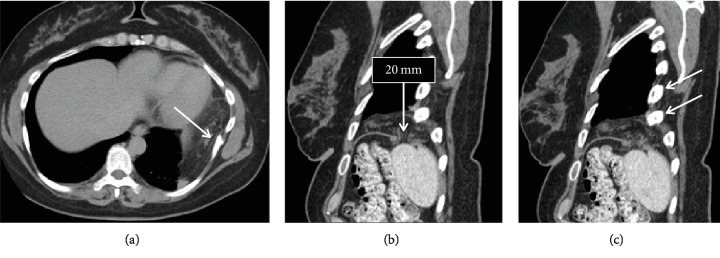
Axial (a) and sagittal (b and c) CT scan images showing diaphragmatic omental herniation. (a) also shows a healed rib fracture (arrow), which is adjacent to the herniated omentum. Two healed rib fractures are also indicated by arrows in (c).

Nearly 20 years after the MVA (10 years following orthopaedic consultation), the patient developed unpredictable episodes of sharp left-sided “gassy” abdominal pain and nausea. These episodes occurred 2-3 times per week, lasting 3-24 hours, and would resolve after vomiting or dry heaving. The patient denied associated fevers, chills, or diarrhea. The frequency and duration of these nausea spells also increased over the following two years. A work-up for gallbladder and hepatobiliary problems was negative (but no abdominal CT scan was ordered). Plain radiographs taken at an urgent care clinic in January 2018 showed findings suggestive of a left-side diaphragmatic hernia. A general surgeon (D.G.M.) was consulted, and a CT scan was done that confirmed the diagnosis of a left-sided, posttraumatic diaphragmatic hernia with a defect measuring 2-2.5 cm in the anterior-lateral left diaphragm juxtaposed to healed rib fractures. There were abdominal contents including omentum in the left pleural space (Figures 1 and 2).

The patient was then referred to another general surgeon specialist (R.E.G.) for laparoscopic repair. During laparoscopy, the hernia containing incarcerated omentum was visualized in the left anterior-lateral diaphragm. The omentum was reduced into the abdominal cavity. There was a focal adhesion to the lung that was divided through the defect. The diaphragmatic defect was closed primarily using polyester suture with Teflon pledgets incorporated in the stitches, and no mesh was required. A red rubber catheter was advanced into the defect prior to closure. After conclusion of the surgery and deinsufflation of the CO_2_-inflated abdomen, the pleural space was aspirated to reduce the chance of pneumothorax, and the catheter was removed. A chest X-ray in the recovery room confirmed lung reexpansion and no pneumothorax. The patient was discharged on postoperative day #1 with an uneventful recovery. Immediately following diaphragmatic repair, the patient reported complete resolution of all symptoms including shoulder pain, mandibular pain, abdominal pain, and nausea. At 20 months after laparoscopy at her final follow-up appointment, she reported no recurrence of any symptoms.

## 3. Discussion

Acute diagnosis of TDR largely depends on the nature of the concomitant primary injuries, the size of the diaphragmatic defect, and the presenting symptoms [4]. Large TDRs (>10 cm) are associated with blunt force trauma, higher morbidity, and higher mortality, while small TDRs are more often associated with penetration by a displaced fractured rib, stabbing injury, or gunshot [2, 24]. When compared to small TDRs, large TDRs are also more likely to show herniation of abdominal contents into the thoracic cavity with imaging, leading to a more rapid repair [25]. Our patient's TDR was likely caused by penetration from a displaced rib fracture that was missed because it was too small to show signs of herniation. She also had splenic bleeding and a pneumothorax in the vicinity that likely impeded detection of the TDR. While multidetector CT imaging is more sensitive and specific than other imaging modalities for detecting diaphragmatic rupture [26], exploratory laparoscopy affords greater sensitivity and specificity [27, 28] and should be considered for multitrauma patients whose symptoms are not explained by CT results. Had our patient's splenic rupture been treated surgically, the TDR may have been visualized and repaired before any symptoms of shoulder pain began.

Our patient's atypical symptoms of left shoulder and mandibular pain likely also contributed to the delay in diagnosis. Irritation of the diaphragm can cause referred pain along the phrenic nerve, mostly along the C4 dermatome [29], which is a phenomenon known as Kehr's sign [30]. However, this has not been reported as a consequence of TDR. The most common presenting symptoms of diaphragmatic hernia include respiratory distress, dyspnea, shortness of breath, abdominal pain, nausea, and vomiting [6–22]. These symptoms arise when abdominal viscera or omental fat become incarcerated or herniate into the thoracic cavity through the unhealed diaphragmatic defect.

The diaphragm is not known to spontaneously heal, and defects can enlarge over time because the abdominal-thoracic pressure gradient of 9-12 mmHg draws abdominal contents through the defect into the pleural cavity [2, 31]. Our patient's TDR likely grew over time, eventually getting large enough for her abdominal contents to begin herniating into the thoracic cavity. This hernia resulted in episodes of epigastric and abdominal colicky pain, nausea, and vomiting, which progressively became more frequent and severe. At the time of diagnosis, only omental fat was found herniating through the diaphragmatic defect, which is referred to as the “funky fat” sign in CT scan images [5].

Surgical repair is indicated in nearly all cases of TDR. The surgical approach depends on the time of presentation following injury and the size of the defect. Acute presentations are often managed via laparotomy to assess other visceral damage, while delayed presentations with herniating viscera may be managed via thoracotomy to address intrathoracic adhesions [32]. Stable patients can be managed with laparoscopy, thoracoscopy, or video-assisted thoracoscopic surgery (VATS). Smaller diaphragmatic ruptures can be primarily repaired (as done in our patient), while larger defects may require incorporation of a mesh [32, 33]. Our patient's left shoulder and mandibular pain immediately and completely resolved following repair of the diaphragm, supporting our conclusion that these symptoms were caused by diaphragmatic irritation rather than intrinsic shoulder pathology.

## 4. Conclusion

This report describes the case of a female with a delay in diagnosis of a TDR with eventual herniation from a rib fracture sustained during an MVA 22 years prior. This case is unique because (1) she presented to an orthopaedic surgeon with a chief complaint of chronic shoulder pain, (2) it is the fourth longest delay in diagnosis of TDR that we could locate in our literature review, and (3) the unique symptoms of ipsilateral referred shoulder and mandibular pain were attributed to a diaphragmatic defect. Due to the high morbidity and mortality associated with diaphragmatic hernias, early diagnosis and subsequent repair of TDR are critical. Physicians should recognize that patients with rib fractures and adjacent visceral injuries are at a higher risk for TDRs that might not be revealed on CT scans or present typically. One of these atypical presentations includes chronic shoulder pain that does not improve with standard medical/surgical interventions. This report supports expanding the differential diagnosis to include TDRs in patients with persistent shoulder pain following rib fractures.
